# Understanding electrical traffic jams in the power grid

**DOI:** 10.1038/s44172-022-00028-1

**Published:** 2022-11-03

**Authors:** Miranda L. Vinay

**Affiliations:** Communications Engineering, https://www.nature.com/commseng

**Keywords:** Energy science and technology

## Abstract

In a recent work published in *Nature* *Communications*, Dr. Benjamin Schäfer and colleagues demonstrate the effect of Braess’ paradox in power grids, both in a lab-scale mimic and through real-world simulations of the German power network. The results lay the groundwork for more sustainable grid development.

Power grids are a fundamental component of modern infrastructure. However, expanding them to integrate renewable energy sources comes with risks that are not fully understood. Previously, theoretical mechanisms predicted that adding transmission lines may hurt the greater grid performance, potentially leading to blackouts. This is known as Braess’ paradox^1^. But so far, the theory lacked experimental validation on a realistic scale.

Braess’ paradox is a mathematical phenomenon from traffic modelling. It states that adding capacity to a network (or lanes of traffic) may reduce the overall system performance, as additions to the network put extra stress on other weak spots. It can be found in ecological systems, team sports strategies, and the power grid. In grids, increasing power transmission capacity by adding new lines could destabilize the whole system.

Experimentally showing a mathematical paradox is not easy. The theoretical groundwork for this study was published in 2012^1^, and efforts for experimental demonstrations have been under development since. In the lab, Dr. Benjamin Schäfer and colleagues built an exploratory platform made from generators, motors and power lines to mimic a simple power grid. They found that upgrading one line to increase the transmission of power rerouted the flow of electrical current. This redirection may be towards lines which weren’t prepared for increases in flow and could overload an unsuspecting transmission line. Once a line is overloaded, it becomes prone to overheating or tripping. Thus, upgrading the line created a case of Braess’ Paradox.

The authors then asked the question, in a more complex network, could they predict a Braessian edge? In other words, where in a power grid does the maximally loaded line become overloaded as a result of extension? They introduced a topological criterion to predict how the modification of one part of the network would affect flow elsewhere. The criterion was successfully applied in the prediction of Braessian edges in different scenarios, including the IEEE 300 bus test case, a simple approximation of an Electric Power system.

**Figure:**
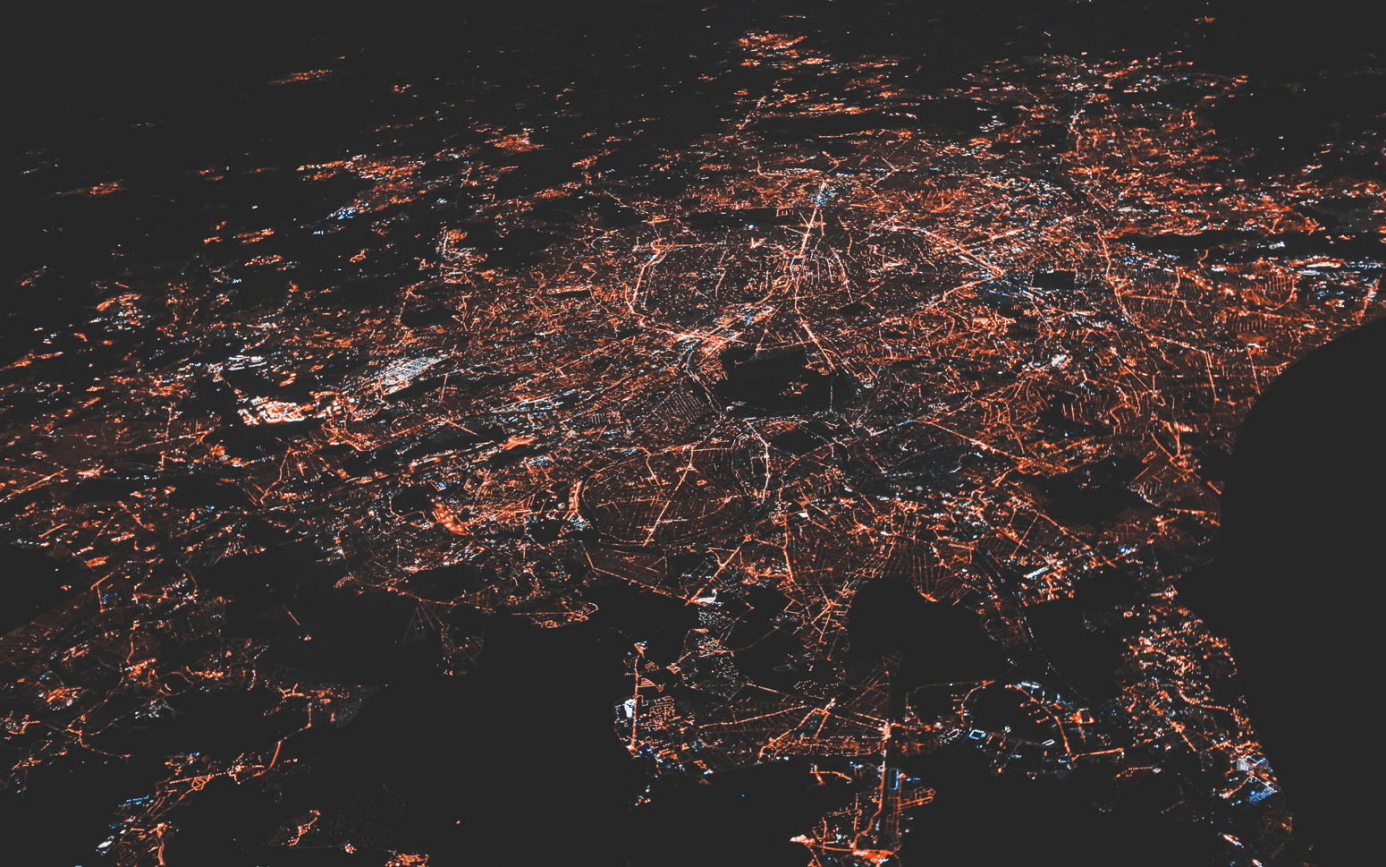
Photo by Nastya Dulhiier on Unsplash

The researchers also sought to characterize real systems. They gathered information from the German high-voltage power grid and its extension plan, called the Netzentwicklungsplan (NEP)^2^. The researchers found that under the highest wind power injection scenarios, two particular grid extensions already under construction have resulted in an emergence of Braessian edges. Both measures effectively relieve grid load in one part of the network but increase loads elsewhere. The findings highlight the need for systemic grid extension planning and broad network reinforcement beyond single-line extensions. Understanding Braess’ paradox in grid systems could help prevent blackouts and ease the integration of new energy technologies.

Dr. Schäfer remarks, “The most difficult challenges were coordinating the diverse and interdisciplinary team of authors: People from quite different backgrounds collaborated on this project (experimental engineering, computational engineering, theoretical physics). In addition, the project took so long to finish that multiple authors moved to a new institution (and sometimes different countries) or retired.” However, after five years of research, the findings were finally published in *Nature Communications*.

This work settles the debate on if and how Braess’ paradox emerges for real-world power grids, both at the lab scale and in lifelike simulations. But it goes further. “We wanted to provide intuitive explanations and guidance for power grid operators,” recalls Dr. Schäfer. To prevent failures, the researchers recommend that engineers consider shutting down working lines to decrease transmission load and thereby improve overall grid stability. Additionally, the authors provide a tool to identify which lines need to be disconnected in order to maintain stability.

Now that Braess’ paradox has been found in real systems, the researchers are eager to dive into the next experiments. “We are quite curious to discuss this further with transmission system operators and other companies and government agencies responsible for maintaining a stable power grid,” Dr. Schäfer said. Power companies can rejoice: “I do believe planning and operation of grids could profit if this effect was widely known and ideally considered in the planning of grid extensions.” And energy consumers can look forward to more reliable services as the grid changes.

The original article can be found here:

Schäfer, B. et al. Understanding Braess’ Paradox in power grids; *Nature Communications* **827**, 5396 (2022), 10.1038/s41467-022-32917-6

The authors have also written a *Behind the Paper* for the Nature engineering community website, which can be found here:

https://engineeringcommunity.nature.com/posts/adding-only-the-right-lines-in-energy-networks?channel_id=behind-the-paper.
